# Data on healthcare perceptions about system risk factors associated with patient safety from the Ministry of Health hospitals in Hail Region of Saudi Arabia

**DOI:** 10.6026/97320630017274

**Published:** 2021-01-31

**Authors:** Fares Alshammari, Hamoud Fahad Alshammari, Bandar Alsaedi, Rafat Zreiq, Fahad D Algahtan

**Affiliations:** 1 Department of Health Informatics, College of Public Health and Health Informatics, University of Ha'il, Ha'il, Saudi Arabia; 2 Department of Health administration, College of Public Health and Health Informatics, University of Ha'il, Ha'il, Saudi Arabia;; 3 Department of Public Health, College of Public Health and Health Informatics, University of Ha'il, Ha'il, Saudi Arabia; 4 Molecular Diagnostic and Personalised Therapeutics Unit, University of Ha'il, Ha'il,Saudi Arabia

**Keywords:** Patient safety, medical errors, system factors, Saudi Arabia

## Abstract

Patient protection has become one of the key elements of the quality of health care systems in Saudi Arabia. Medical errors that threaten patient safety are mediated by several factors including system risk factors. Hence, we used a self-structured questionnaire to assess and rank the system factors according to the perceptions of nurses working in the hospitals of the ministry of health in Hail, KSA. Eight out of twelve factors tested were perceived as threatening factors of the patient safety that are; 'Shortage of medical staff', 'Poor design of the hospital structure', 'Long working hours', 'Overcrowding of patients','Poor coordination between hospital departments, 'Punitive and blaming environment, 'Lack of clinical practice standards' and, 'Poor financial incentives'. Thus, considering the negative impact of the identified threatening system factors in this study on patient safety, urgent planning and managing appropriate corrective actions should be designed to improve patient safety issues.

## Background

Although the delivery of healthcare services has improved significantly, the number of reported serious medical errors (MEs) continues to rise throughout the world. The problem of MEs is still a global phenomenon that harms patients, families, and healthcare systems and it is the leading cause of iatrogenic unfavourable outcomes in the healthcare industry. MEs are primarily under the management of health care professionals and patients. A ME is defined as a failure to accomplish planned actions or the use of incorrect plans (either of commission or omission) to achieve an intended purpose and subsequently leading to disability and death. Patient safety is a global problem, which affects rich and poor countries alike. In this context, studies have shown that MEs and adverse drug reactions are one of the major causes of adverse effects resulting in illness and death in hospitals, reflecting up to 6.5% of hospital admissions [1-4]. According to Donaldson and Philip, medical errors and adverse events affect 10% of patients during the course of treatment in the developed countries [5]. In the US, between 44,000 to 98,000 patients die every year because of incidents of medical error, which exceeds the number of deaths due to motor vehicle accidents, breast cancer, and AIDS [6]. The annual number of preventable adverse events in the United Kingdom is estimated to be 400,000; resulting in over 34,000 deaths per year (Hunt 2004), which far exceeds the number of deaths from motor vehicle, workplace, and aviation accidents [7]. In Australia, medical errors result in as many as 18,000 preventable deaths and more than 50,000 disabilities among patients every year [8]. The magnitude of medical errors in the developing countries is greater than in the developed world [9], which is due to weak infrastructure and poor equipment, poor quality and supply of drugs, poor procedures of infection control, unreliable medical practice, and the lack of financial resources [10]. In Saudi Arabia, Ministry of Health (MOH) receives 40,000 complaints about incidents involving medical errors every year. Further investigations of these complaints, 20% (8,000 cases) have been proven to be actual medical errors. However, the rate of medical errors that is reported by the MOH does not reflect the real magnitude of the problem in the Saudi Healthcare System, since a large proportion of medical errors is not documented, especially in rural areas. MEs happen is a multi-factorial that may occurs at any time in the remedy process, from ordering to medication intake by the patients [11], [12]. These factors are mainly categorized into three dimensions including system factors, patient factors and human factors [13-19] Distinct from other healthcare professionals, nurses play an important role in discovering the causes of medical errors due to the nature of their work which includes direct communication and accompanying patients for long periods, making them the primary link between patients and other healthcare professionals. Therefore, nurses’ perception toward factors associated with medical errors is vital. It is of interest to identify the perceptions of nurses regarding the system risk factors that undermine MEs in hospitals located in Hail city, run by the MOH, Saudi Arabia. The objectives of the present study are (i) to investigate the perceptions of nurses regarding system risk factors that undermine MEs, (ii) to rank the system risk factors and (iii) to assess differences among characteristics of the study sample in relation to the system risk factors.

## Methodology

This research was designed as a descriptive study. A cross-sectional survey, using self-administered questionnaires, was used for data collection. Three hospitals located in Hail city i.e. King Khalid Hospital, Hail General Hospital and Maternity and Children's Hospital were selected using cluster sampling method. The permission of these hospital authorities has been obtained to conduct this study. A structured questionnaire method consisting of two sections were distributed to 450 nurses working in the selected hospitals. The first section covered the characteristics and demographics of the participants while the second section investigated nurses' perceptions toward the diminution of system risk factors causing MEs. Only 246 questionnaires contained complete information on key variables and used for analysis. The overall response rate was 54.66% (n=246).

## Validity and reliability:

The study assessed the validity of the questionnaire before undertaking the process of data collection. Both, face validity and content validity were assessed according to [20]. The value of Cronbach's alpha of the scale was considered reliable since the alpha value was greater than 0.80 for the system risk factors scale (0.85).

## Data Analyses:

Data were analysed using the SPSS Statistics 22.0 (IBM SPSS Statistics, New York, NY, USA). The level of statistical significance was set at p < 0.05. Descriptive statistics (means, standard deviations (SD), and frequencies) were presented for the studied variables. The system risk factors were ranked according to the mean of the whole scale by applying Friedman test [21]. The Chi-Square and the significance level (P-value) indicate if there is a statistically significant difference in the rank between these factors. Since there were 12 factors listed in the second part of the study questionnaire, the rank ranged from one to 12.

## Results:

### Demographic characteristics:

Six demographic variables were included in the survey questionnaire to identify the background characteristics of the respondents. These characteristics include age, gender, nationality, medical department, work experience and professional level. The variable 'age' was classified into 4 groups; less than 20 years, 20 to 29, 30 to 39 and ≥ 40 years old. Out of the total respondents, more than fifty per cent (i.e. 58.1%) of the respondents were within the age group of 20 to 29 years (Table 1 - see PDF). The data also showed that most (98.4%) of the respondents were female. In addition, more than 50% of the respondents were Saudis citizens (57.3%), whereas resident's nurses constituted 42.7% of the study sample.

The variable "medical department" describes the current department in which the study participants are working. Medical department was classified into six groups; obstetrics and gynaecology, internal medicine, emergency room, Paediatrics, surgery and others. The analysis revealed that most of the respondents (43.5%) belong to obstetrics and gynaecology department (Table 2 - see PDF). The variable "work experience" was categorised into four groups; less than 5 years, 5 to 9 years, 10 to 14 years, and 15 years and more. The data showed that almost half of the respondents (49.2%) had less than five years of work experience (Table 2 - see PDF). The variable "professional level" was divided in to three categories: nursing technician (Diploma), registered nurse (RN) with a Bachelor degree, and others (advanced certificates). The data showed that nursing technicians represented over half of the study sample (54.9%), followed by 42.7% for RN, and 2.4% for other nursing professionals (Table 2 - see PDF).

### System risk factor:

In the second part of the survey questionnaire, nurses were asked to quantify the system risk factor that may exist in their hospitals in relation to medical errors. Respondents' perceptions of the existence of the each of the system risk factor in the MOH hospitals are presented in Table 3 (see PDF). Moreover, factors were ranked to the mean value. Among the top risk factors (mean >12.11), eight items were considered as strong system factors (Table 3 - see PDF). System factors cited by the study respondents included: 'Shortage of medical staff', 'Poor design of the hospital structure', 'Long working hours', 'Overcrowding of patients', 'Poor coordination between hospital departments', 'Punitive and blaming environment', 'Lack of clinical practice standards' and, 'Poor financial incentives'.

### Differences among characteristics of the study sample in relation to the system risk factors:

#### Age:

Table 4(see PDF) shows the Correlation between nurse's age and the system risk factors using Spearman rank correlation analysis 'poor coordination between hospital departments' and 'lack of clinical practice standards' showed statistically significant (P<0.05) positive correlation with age, suggesting that these factors were perceived to exist in the MOH hospitals of the Hail region by older nurses more than their younger counterparts. In contrast, 'Long working hours' had statistically significant negative correlation with age, indicating that this factor was perceived to exist by younger nurses rather than their older counterparts. No significant correlation was detected for other factors with age. To assess the differences between male and female nurses, and between Saudi and non-Saudi nurses in relation to the system factors, the Mann-Whitney test was carried out. There was a statistically significant (P<0.05) difference between male nurses and their female counterparts in relation to only one factor 'long working hours'. The higher mean rank for female nurses indicate that this factor was perceived to exist in the MOH hospitals by female nurses more than their male counterparts (Table 5 - see PDF). Table 10 (see PDF) shows there were statistically significant differences between Saudi nurses and their non-Saudi counterparts in relation to two risk factors i.e. 'long working hours' and 'poor coordination between hospital departments'. Saudi nurses, with higher mean ranks, perceived a higher risk of the shortage of medical staff than their non-Saudi counterparts did. Non-Saudi nurses, with higher mean rank, emphasized on the poor coordination between hospital departments in their hospitals.

#### Medical department:

Results of the Kruskal-Wallis test found that there were significant differences among nurses across different medical departments in relation to their perception of shortage of medical staff, long working hours, and overcrowding of patients (Table 7 - see PDF). Other departments, pediatric nurses and emergency nurses, perceived a higher risk factor of shortage of medical staff, long working hours, and overcrowding of patients respectively. Interestingly, surgery nurses emphasized a lower risk of all these three factors.

#### Work experience:

Results of the Spearman rank correlation test showed that there was a significant negative relationship between the variable "work experience" and the risk factor "long working hours" (Table 8 - see PDF). Nurses with shorter working experience reported that there was long working hours in the hospital more than those of longer experienced nurses. In contrast, we found that there is a significant positive relationship between respondents' work experience and the risk factor 'poor coordination between hospital departments'. Nurses with longer experience reported that there was a poor coordination between hospital departments than the shorter experienced nurses.

#### Professional level:

To explore the association between respondent's professional level and various system-level risk factors, the Spearman rank correlation analysis was undertaken (Cooksey 2007:106). We found that there is a statistically significant positive association between the professional level of nurses and five system factors: 'shortage of medical staff', 'overcrowding of patients', 'poor coordination between hospital departments', 'punitive and blaming environment', 'lack of clinical practice standards and guidelines' (Table 9 - see PDF).

## Discussion:

This study highlights the significance of patient safety from the viewpoint of nurses in MOH hospitals located in Hail region. The study showed that patient safety was perceived by the respondents to be violated by several system factors. The majority of nurses (42.2%) perceived moderate overall system risk factors, followed by 36.8% who perceived strong overall system risk factors. Eight factors were considered as strong system risk factors as the mean score of these factors is above the mean score of the overall mean of the scale (mean >12.11). These system risk factors were: 'Shortage of medical staff', 'Poor design of the hospital structure', 'Long working hours', 'Overcrowding of patients', 'Poor coordination between hospital departments', 'Punitive and blaming environment, 'Lack of clinical practice standards' and, 'Poor financial incentives'. According to the nurses' perceptions, other system risk factors had weak influence on patient safety in the hospitals they are affiliated to. These factors were; outdated medical equipment, insufficient continuous medical, education and training for medical staff, and shortage of medical supplies Shortage of drugs. Our study showed, as perceived by the respondents, that the 'shortage of medical staff" is the riskiest factor in the system which negatively affecting patient safety in the MOH hospitals in Hail. Our findings are inconsistent with the results of other studies. For instance, the shortage of staff to handle the workload has been reported as the highest mean value received among other factors [22]. Shortages in medical staff, in particular nurses, have serious consequences and this can be attributed to the massive turnover rate of nurses [23]. In this context, Cimiotti et al. (2012) reported a significant positive association between nurse burnout and poor quality care, especially in infection rate and mortality rate, in the US hospitals [24][25]. Alenius and colleagues argued the deficiency of patient safety practices due to patient-to-nurse ratios [26]. Additionally, the shortage of nursing staff definitely increases the workload of the nursing staff and results in high pressure on nurses, which is in turn considered a major cause of errors [27], [28]. Thus, our findings support other studies in the role of medical staff sufficiency as a crucial factor for achieving patient safety. Factors-demographic characteristics correlations revealed that the 'shortage of medical staff" showed a significant positive association with working in other medical departments and professional level of the respondents, but less those working in the surgery department. In the second rank, poor design of the hospital structure is also a serious risk factor in the system. Examples of poor design of the hospital structure are weak designed facilities, technology and equipment [29]. The negative impact of long working hours is well reported in many studies as one of the most system risk factor associated with patient safety, which is in agreement with our finding [22], [30]. Moreover, these studies reported no significant correlations between long working hours with any of the demographics characteristics. In contrast, our study showed significant positive statistically significant positive correlations with gender, nationality, and working departments but negatively with age, surgery, and Experience. These differences might be due to temporal and spatial variations among these studies, which lie under the limitation of this study. Hospital overcrowding remains a major obstacle in front of patient safety worldwide. Patients overcrowding and long waits, in particular in emergency departments, are considered as leading causes of high mortality [31]. In this context, it has been demonstrated that proper administration of patient overcrowding enhances patients' perceived safety. Our study reported patients crowding as one of the most important factors affecting patient safety and, in agreement with Epstein study, reported the perception of nurses from the emergency department with the highest mean score for this factor. According to the perception of the nurses' participants, the risk factor 'Punitive and blaming environment' also received a high mean score in the system risk factors scale. This is in agreement with the finding reported by Al-Ahmadi, in which non-punitive response to an error received the lowest positive response [27]. Finally, 'lack of clinical practice' was also received a higher mean score in the system risk factors scale. This implies that incorporating nurses in more clinical practice programs are perceived to enhance patient safety. Our results showed that lacking clinical practice has a significant positive association with age as well as professional level. This means that younger and less-professional nurses were more concerned about the lack of clinical practice as an important risk factor than senior nurses were. In this regard, many studies made the argument that clinical experience has a positive impact on the nurse's response in assessing the patient and subsequently positively impacting the patient safety [32],[33]. Although some of the risk factors were ranked lower compared with others, attention to and concern for all risk factors is needed by policy makers and hospital managers in order to improve patient safety in the healthcare settings. The findings of the study may have implications for improving healthcare delivery in the MOH hospitals. The study contributes to the knowledge of patient safety in Saudi Arabia and it offers some insights into the relationship between improving patient safety and factors that might hinder such improvement. Patient safety is an integral part of healthcare delivery, and achieving an acceptable standard of patient safety requires that all levels of a healthcare organization develop a common patient safety system, including both a positive culture of safety and the organizational support for the processes. However, the study does have some limitations. Data collection for the current study was conducted at a fixed point of time; from December 2007 to February 2008. It is plausible that certain internal events in MOH hospitals could have influenced the results. The current study was limited to the MOH hospitals in one geographical area (i.e. Hail region) due to time constraints and the limited resources of project; hence, the proposition that the study results are generalizable across the MOH needs to be investigated through further research. It is also important to note that safety is not just the domain of nurses. In the present study, inclusion of more than one group would have required a much larger sample with, consequently, the need for more time and more resources that were beyond the scope of the project. The future studies may look at the perceptions of other healthcare workers in other regions of the Saudi Arabia. In conclusion, patient safety in the Ministry of Health hospitals in Hail Region of Saudi Arabia suffers from many of system factors. Therefore, urgent planning and managing appropriate corrective actions should be designed and implemented to improve patient safety issues.
